# A Survey of Deep Anomaly Detection in Multivariate Time Series: Taxonomy, Applications, and Directions

**DOI:** 10.3390/s25010190

**Published:** 2025-01-01

**Authors:** Fengling Wang, Yiyue Jiang, Rongjie Zhang, Aimin Wei, Jingming Xie, Xiongwen Pang

**Affiliations:** 1 School of Artificial Intelligence, South China Normal University, Foshan 528000, China; wfl314159@163.com (F.W.); jiangyy@m.scnu.edu.cn (Y.J.); 2 School of Computer Science, South China Normal University, Guangzhou 510555, China; zrj22127@gmail.com; 3 School of Architectural Engineering, Guangzhou Panyu Polytechnic College, Guangzhou 511483, China; weiam@gzpyp.edu.cn; 4 Doctoral Workstation, Guangdong Songshan Polytechnic, Shaoguan 512126, China

**Keywords:** anomaly detection, deep learning network, multivariate time series

## Abstract

Multivariate time series anomaly detection (MTSAD) can effectively identify and analyze anomalous behavior in complex systems, which is particularly important in fields such as financial monitoring, industrial equipment fault detection, and cybersecurity. MTSAD requires simultaneously analyze temporal dependencies and inter-variable relationships have prompted researchers to develop specialized deep learning models to detect anomalous patterns. In this paper, we conducted a structured and comprehensive overview of the latest techniques in deep learning for multivariate time series anomaly detection methods. Firstly, we proposed a taxonomy for the anomaly detection strategies from the perspectives of learning paradigms and deep learning models, and then provide a systematic review that emphasizes their advantages and drawbacks. We also organized the public datasets for time series anomaly detection along with their respective application domains. Finally, open issues for future research on MTSAD were identified.

## 1. Introduction

Time series anomaly detection is crucial in data analysis, focusing on identifying unusual patterns in time series data that significantly differ from expected behavior [1,2]. This task becomes more intricate in MTSAD, which it involves multiple channels or variates of data. The evolution of sensing technologies and enhancements in data storage have propelled the adoption of anomaly detection across a spectrum of sectors. For example, In the financial services sector, it is used in financial services to identify fraudulent transactions and market fluctuations, protecting customer funds [3]. In the industrial sector, it monitors equipment status to detect faults early, reducing downtime and maintenance costs [4].

Multivariate time series anomaly detection has been studied in a variety of application domains and has experienced significant progress. There exist various methods proposed for MTSAD, including statistical methods, classical methods, distance-based methods [5], distributional-based methods [6], and density-based methods [7], are still a viable choice of algorithm. Statistical techniques typically involve moving averages(MA) and the autoregressive integrated moving average (ARIMA) [8]. MA reduce the impact of random fluctuations by calculating the average value within a specific time window, thereby revealing trends and patterns in the data. The ARIMA model is a popular time series forecasting method that combines three components: AutoRegressive (AR), Integrated (I), and MA, which are used to model and predict time series data. Classical machine learning methods include One-Class Support Vector Machine (OCSVM) [9] and Support Vector Data Description (SVDD) [10]. SVDD is similar to OCSVM, but instead of finding just a boundary, it creates a hypersphere that contains the normal data. Any data points outside this hypersphere are considered anomalies. Distance-based techniques assess the deviation of observations from representative data points using distance metrics, while distributional methods focus on identifying anomalies through points with low likelihood. Density-based methods are based on the local density of data points. If a data point has significantly lower local density compared to its neighboring area, it may be flagged as an anomaly. However, as target systems grow in size and complexity, these methods encounter challenges, particularly their limitations in handling multidimensional data and the lack of labeled anomalies [11]. The challenge of MTSAD lies in the need to consider both the dynamic changes along the temporal dimension and the interrelationships between observations simultaneously. Recently, deep learning-based techniques have advanced the field of anomaly detection within multi-dimensional datasets. These methods exploit powerful deep learning models like Transformers [12,13,14], GNN [15], VAEs [16], GANs [17], Diffusion [18] etc., are adept at representing intricate, non-linear relationships among various sensors and are proficient in capturing temporal correlations and ependencies effectively [19]. These advanced models leverage the capabilities of deep learning to identify and learn subtle patterns in data, enabling accurate identification and early warning of anomalous behaviors across various fields such as financial transaction monitoring, cybersecurity threat detection, industrial equipment maintenance forecasting, and healthcare monitoring. The progress of these technologies allows us to better understand and analyze the complex dynamics within multi-dimensional datasets, providing powerful tools for real-time anomaly detection and decision support systems.

In this paper, we aim to address the current knowledge gap by exploring features of time series, types of anomalies, and offering a thorough overview of recent advancements in deep learning techniques for MTSAD. It reviews the latest deep learning methods for this purpose from three perspectives and offers a comprehensive discussion about the classification of deep learning models. Additionally, it compiles and organizes public datasets for time series anomaly detection, along with their respective application domains. Finally, the paper wraps up by highlighting possible directions for future research to further develop the field.

In summary, the contributions of this paper include:

(1) The investigation and review of anomaly categories in multivariate time series have proposed a new classification;

(2) Focusing on learning paradigms and neural network architectures, we conducted a comprehensive review of the latest deep learning methods from three strategies and proposed a new taxonomy for MTSAD;

(3) An exploration of future research opportunities for MTSAD.

## 2. Background and Preliminary

### 2.1. Problem Definition

MTSAD refers to the process of identifying anomalous behavior or patterns that deviate significantly from historical patterns within the context of multiple related time series. Compared to univariate time series anomaly detection, multivariate anomaly detection is more complex as it requires consideration of the relationships and temporal dependencies between multiple variables simultaneously [20]. Anomaly score is an indicator used to measure the degree of anomaly of a time point or time windows. The higher the anomaly score, the more likely the data point is to be anomalous.

We consider a collection of MTS denoted as *X*. Hence, the definition can be expressed as follows:

(1) $$X=(x^{1},x^{2},\ldots ,x^{C})$$

(2) $$x^{c}=\left(x_{1},x_{2},\ldots ,x_{T}\right)$$

where $x^{c}\in R^{T}$ represents an T-dimensional vector, each data point $x_{t}\in R$ is acquired at a certain timestamp *t* from a sensor. *T* represents the length of lookback windows and *C* represents the number of dimensions or variables (C>1).

Given a multidimensional time series dataset $X\in R^{TXC}$, the goal is to find a method to compute anomaly scores:

(3) $$S=(s_{1},s_{2},\ldots ,s_{T})$$

where $s_{t}\in R$ denotes the anomaly score at time point t. This allows us to identify anomalous data points that deviate from normal behavior.

### 2.2. Anomaly Types in MTS

**Figure 1:**
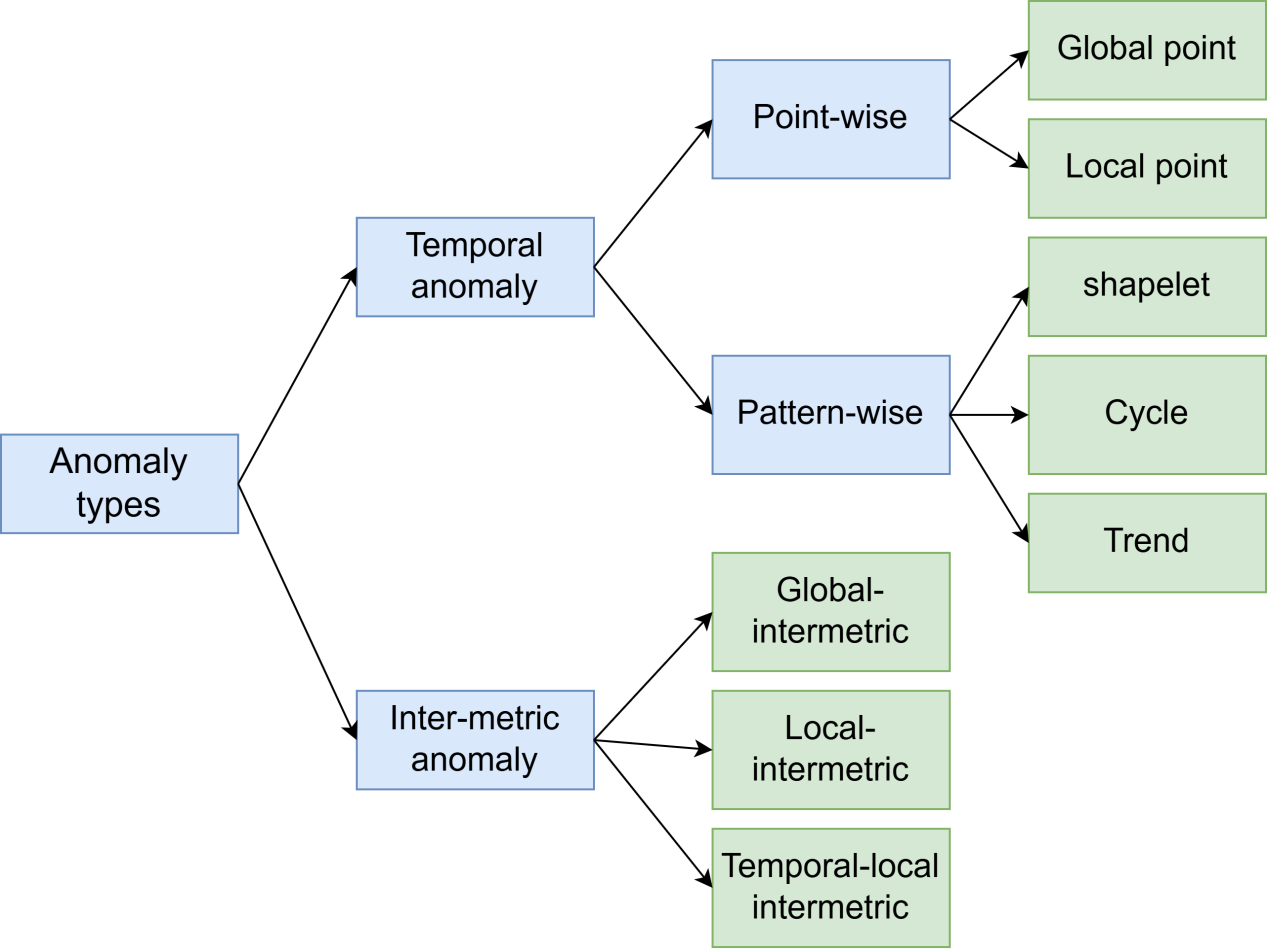
The overall classification framework diagram for multivariate time series anomaly types.

MTS consists of a set of univariate time series(metric), each of which describes different parts or attributes of a complex entity. Therefore, it not only has temporal dependencies within the metrics, manifested as periodic, trend, and other inherent patterns of each metric, but also inter-metrics dependencies within the entity, representing the linear or nonlinear relationships between all metrics of the entity at each time point [2,21]. Thus, anomalies in MTS can be divided into intra-metric anomalies (temporal anomalies) and inter-metric anomalies. The overall classification framework for anomaly types is shown in Figure 1. Temporal anomalies occur in multivariate, affecting multiple or all dimensions. Temporal anomalies primarily focus on individual data points and subsequences. Point-wise anomalies refer to unexpected events at a single time point, where the anomalous behavior of a time point can be a spike or glitch. Pattern-wise anomalies are anomalous subsequences, usually discordant or disharmonious. Here are the definitions of types of anomalies:

#### 2.2.1. Point-Wise Anomalies

Global point anomaly: Global point anomaly is an anomaly in which a single data point is significantly different from other data. They are typically spikes in the entire sequence. Considering a threshold δ, it can be described as:

(4) $$|x_{t}-{\hat{x}}_{t}|>\delta$$

where $x_{t}$ is expected value and ${\hat{x}}_{t}$ is the output of the model, $\delta =\lambda *G(x_{t})$, *G* represents the variance of the context over $x_{t}$, and λ is the threshold adjustment coefficient.

**Figure 2:**
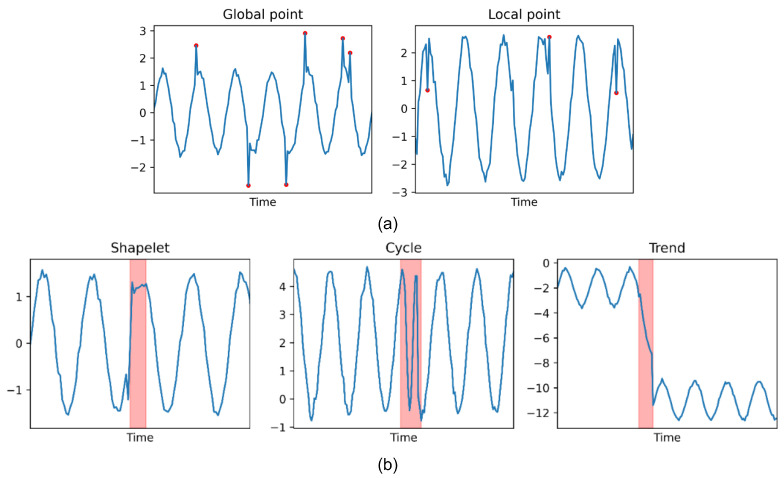
(**a**) Point-wise anomalies, the red dots indicate anomalies, and (**b**) Patten-wise anomalies, the red areas represent anomalies.

Local point Anomaly: Local point anomaly refers to abnormal situations that occur within specific environments or contexts. Detecting local point anomaly typically involves analyzing both the point and its associated contextual information to identify behavior that deviates from the expected norms within a given context. This type of anomaly can be defined as:

(5) $$\delta \approx \lambda *L(x_{t-k}:x_{t+k})$$

where $x_{t-k}:x_{t+k}=\left(x_{t-k},x_{t-k+1},\ldots ,x_{t+k}\right)$ refers to the context of the data point $x_{t}$ with a window size *k*, *L* represents the variance of this local context. An example of point anomalies are shown on Figure 2a, global point anomalies typically stand out against the backdrop of the entire time series, while local point anomalies contrast more with the data points immediately adjacent to them.

#### 2.2.2. Pattern-Wise Anomalies

Shapelet anomaly: Shapelet anomalies are circumstance that occurs in a particular environment or context. Unlike individual data point anomalies, shapelet anomaliy indicates that the collective behavior of a group of related data significantly deviates from the expected or normal pattern, as shown in Figure 2b.

(6) $$diss_{p}\left(p,\hat{p}\right)>\delta$$

where $\hat{p}$ specifies the expected shape of the subsequence. diss is a function that measures the difference between two subsequences.

Trend anomaly: Trend anomalies focus on sudden changes or deviations from the long-term trend in the data. These anomalies may result from sudden events, systemic changes, or other factors leading to abrupt shifts or abnormal growth or decline in the trend direction.

(7) $$diss_{\tau }\left(\tau ,\hat{\tau }\right)>\delta$$

where $\hat{\tau }$ is the trend of normal subsequences.

Cycle Anomaly: Cycle anomalies occur when there are abnormal changes in the periodic patterns of time series data. For example, there is anomaly in the frequency or intensity of a seasonal event.

(8) $$diss_{s}\left(s,\hat{s}\right)>\delta$$

where $\hat{s}$ is the cyclicity of expected subsequences.

#### 2.2.3. Inter-Metric Anomalies

**Figure 3:**
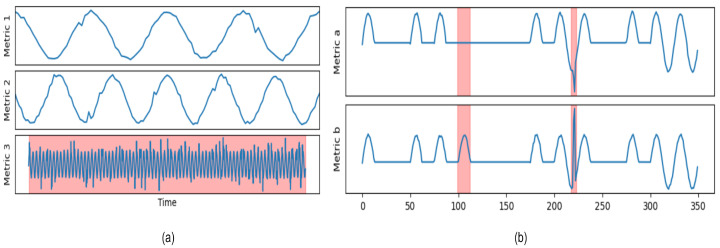
(**a**) Global intermetric anomalies. (**b**) The red-highlighted area on the left indicates local intermetric anomalies, while the red-highlighted area on the right indicates temporal-local intermetric anomalies.

Global intermetric anomaly: Certain metrics in MTS experience significant changes, with a length matching the entire sequence. This type of anomaly typically occurs when initial parameters or system states deviate from the normal range. When the change in a key metric triggers a series of abnormal fluctuations in related metrics, the entire sequence reflects this anomaly. An example of an entire sequence anomaly is shown in Figure 3a, as its behavior significantly deviates from that of other metrics.

(9) $$diss_{corr}\left(corr\left(x^{i},x^{j}\right)\right)>\delta$$

where $x^{i}$, and $x^{j}$ are different metrics, and corr represents the degree of correlation between them.

Local intermetric anomaly: There is correlation between metrics within the entity, which can be linear or nonlinear, such as the relationship between temperature and electricity consumption. If these correlations are disrupted, MTS will exhibit anomalous behavior. During a certain time period, the relationships between metrics significantly deviate from historical patterns, suggesting a change in the interdependencies among the metrics. For example, in Figure 3b, there is initially a positive correlation between two metrics, but the highlighted red area on the left clearly breaks this relationship, leading to anomalies.

(10) $$diss_{corr}(corr\left(x^{i},x^{j}\right),corr\left(x_{t:t+k}^{i},x_{t:t+k}^{j}\right)>\delta$$

where *k* is the time window. If the correlation is disrupted from *t* to t+k, it indicates that the correlation coefficient has exceeded the threshold compared to the normal value.

Temporal-local intermetric anomaly: The anomalies violate the dependencies between metrics and temporal relationships, focusing on how a specific metric breaks its correlation and deviates from its historical trend within a certain time period, as indicated by the highlighted red area on the right side of Figure 3b.

### 2.3. Time/Frequency Domain Analysis

Time series frequency domain analysis involves transforming a time domain signal, which is a signal that varies over time, into a frequency-domain representation. This conversion allows for a better understanding of the signal’s frequency components and periodicity. Frequency domain analysis reveals the different frequency components within a time series, which helps in identifying cyclic patterns, trends, and anomalies. The most commonly used formula is the Discrete Fourier Transform (DFT):

(11) $$X_{k}=\sum_{t=0}^{T-1}x_{t}e^{-i2\pi kt/T},\;k=0,\ldots ,T-1$$

where $X_{k}$ is the discrete complex amplitude at frequency *k*, $x_{t}$ is the time series signal, and *i* is the imaginary unit.

The Fast Fourier Transform (FFT) typically uses Formula (11) to convert the time domain into the frequency domain. FFT is an efficient computational algorithm for the DFT, it can convert the original time series into a spectrum and sort it by coefficients, obtaining the seasonal cycle by reversing the highest frequency. Most programming languages and mathematical libraries provide built-in FFT functions that can be directly used to compute the DFT.

## 3. Taxonomy of MTSAD Methods

**Figure 4:**
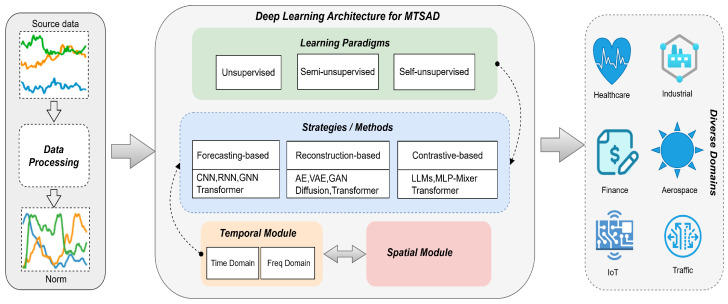
The general pipeline for MTSAD using deep learning models. Given a source data, we first process the source data using a data processing module that performs basic data cleaning and normalization tasks. Subsequently, we utilize the anomaly detection strategies from the perspectives of learning paradigms and deep learning models to obtain representations for executing anomaly detection tasks across different application domains.

To summarize the existing deep multivariate time series anomaly detection methods, we proposed a taxonomy for the anomaly detection strategies from the perspectives of learning paradigms and deep learning models. We present a general pipeline as illustrated in Figure 4. We categorize anomaly detection methods into forecasting, reconstruction and contrastive types. A forecasting-based model uses next timestamp predictions, whereas a reconstruction-based model uses latent representations of the whole time series. Contrastive-based methods rely on calculating the similarity or dissimilarity between data points to conduct analysis and prediction. Table 1 offers a more detailed overview of these methods.

### 3.1. Temporal/Spatial

Temporal analysis focuses on how variables change over time, while spatial analysis examines how these variables vary across different locations or spatial units. Temporal and spatial perspectives offer a more thorough understanding of the underlying relationships and patterns in MTS.

### 3.2. Related Learning Paradigms

Unsupervised, semi-supervised and self-supervised methods are common learning paradigms in MTSAD, and they can be distinguished according to the label information of the data and the degree of supervision:

Unsupervised Learning: Unsupervised anomaly detection does not rely on any labeled anomalous data for training. It attempts to discover anomalous patterns by analyzing the features of the time series, which means it needs to use only normal data during the training phase.

Semi-unsupervised Learning: In semi-supervised learning, only part of the training data has label information, while the other part of the sample has no label information, the model uses the sample with label information to learn during the training process, and tries to improve the performance through the unlabeled sample. In the multivariate time series anomaly detection, the semi-supervised learning method can use the labeled anomaly sample the original and unlabeled normal samples are trained to distinguish between normal and abnormal time series patterns.

Self-supervised Learning: Self-supervised methods utilize unlabeled data to train models by creating supervisory signals from the data itself. These methods typically involve predicting missing parts or properties of the input based on the observed data, allowing the model to learn useful representations without requiring explicit labels, which can then be applied to various downstream tasks.

### 3.3. Model Input

The model input of multivariate time series anomaly detection can be time steps or windows (sliding windows).Time steps refer to individual points in time where observations are recorded for each variable in the multivariate time series. Each time step includes measurements for all variables at that specific moment. Window is subsets of consecutive time steps with a fixed size. These windows are used to aggregate data over time, capturing temporal patterns and relationships among variables within each window. In real world, there are often complex structures such as noise, seasonality, trends, etc., and anomalies usually do not appear at a single point in time, but persist or present a pattern over a period of time. Therefore, the most recent MTSAD model takes a window instead of a single point as the model input.

## 4. Forecasting Methods

**Figure 5:**
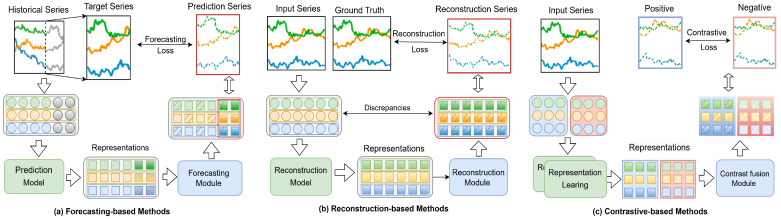
The examples of each type of anomaly criteria: (**a**) a forecasting loss; (**b**) a reconstruction loss; and (**c**) a contrastive loss.

Forecasting-based methods use historical data of time series to train a predictive model that can forecast future data points. This method assesses the anomaly level by comparing predicted values with actual values, considering anomalies as a significant increase in prediction error, as illustrated in Figure 5a. This section will explore predictive anomaly detection methods, focusing primarily on four types: CNN-based, RNN-based, GNN-based, and Transformer-based models.

### 4.1. CNN-Based Models

DeepAnt [22] proposes an unsupervised anomaly detection method based on CNN, which does not require labeled training data. This method uses CNN to automatically learn the representation of time series data and identifies anomalies using reconstruction error. Compared to traditional CNNs, Temporal Convolutional Networks(TCN) can better handle the temporal characteristics of time series, TCN-ms [23] uses TCN for anomaly detection, emphasizing the importance of temporal information. By introducing causal convolution and dilated convolution in the convolution operation to process time series data, it helps to capture long distance dependencies. However, the aforementioned methods only consider the one-dimensional changes of the time series. To enhance the model’s understanding of the structure of time series, TimesNet [24] extends the analysis of temporal changes to 2D space by innovatively incorporates Fast Fourier Transform, making it easy to model 2D changes with 2D kernels, and providing a new perspective for more complex time series analysis. It not only improves the accuracy of anomaly detection but also enhances the model’s generalization ability of the model for time series data.

### 4.2. RNN-Based Models

LSTM-NAT [25] represents an innovative application of RNNs for spacecraft telemetry analysis, emphasizing the use of LSTM [64] to identify anomalies within complex data. Unlike traditional methods, LSTM-NAT employs a non-parametric dynamic thresholding method, utilizing an unsupervised approach to assess residuals and determine whether prediction errors indicate spacecraft anomalies. However, its reliance on one-dimensional outputs limits its ability to handle high-dimensional data in multivariate scenarios. Building upon this foundation, LGMAD [26] integrates LSTM with the Gaussian Mixture Model (GMM) [65], providing a comprehensive solution for low-dimensional system characteristics. This method not only enhances the efficiency of anomaly detection through the health factor but also expands its application to the processing of MTS by leveraging the strengths of both LSTM and GMM in capturing temporal dynamics and analyzing feature correlations. Another notable advancement in capturing temporal dynamics is the THOC [27]. By introducing the Temporal Hierarchical One-Class network, which uses a dilated RNN with skip connections [66], the limitations of LSTM in capturing multi-scale temporal dynamics are addressed. The hierarchical clustering process of THOC and the introduction of a self-supervision task in the temporal domain significantly enhance the model’s ability to detect anomalies in real-world time series data. However, these schemes may require adjustments for specific types of multi-seasonal time series data to ensure their accuracy and efficiency. Wu et al. [28] proposed an unsupervised scheme with multi-seasonality. AD-LTI introduced a new metric—Local Trend Inconsistency (LTI), which can be computed efficiently and robustly scores the probability of anomalies for each data point. It overcomes the challenges of requiring labeled data and the computational intensity of processing large volumes of data, making it suitable for a variety of event-sensitive scenarios.

### 4.3. GNN-Based Models

In the field of MTSAD, capturing the complex relationships and dynamic changes in MTS is crucial. The MTAD-GAT [29] model learns complex dependencies on both the feature and temporal dimensions via a graph attention network [67] and achieves high performance in MTSAD through a joint optimization strategy. However, it may encounter issues with efficiency and scalability when dealing with large-scale datasets. GDN [30] leverages graph neural networks to learn a dependency graph among sensors and enhances the interpretability of anomaly detection through attention weights. However, it fails to fully capture the spatial and temporal correlations between sensors when processing sensor data. To address these issues, Chen et al. [31] proposed DVGCRN, effectively models the spatial and temporal correlations of time series by combining an Embedding-Guided Probabilistic Generative Network(DEPN) with an adaptive variational graph convolutional recurrent network(GCRN), demonstrating superior performance in anomaly detection. FuSAGNet [32] integrates feature selection with GNNs to effectively identify and model the relationships between different time series features. The model constructs an inter-series adjacency matrix based on the latent feature representations generated by a sparse auto-encoder, enhancing its ability to detect anomalies. The DyGraphAD [33] framework strengthens the capture of short-term and long-term dependency relationships in time series through dynamic graph forecasting, thereby improving detection accuracy.

### 4.4. Transformer-Based Models

Transformer-based models, with their self-attention mechanisms, have become a cornerstone in a variety of sequence modeling tasks [12], including time series analysis. These models are particularly adept at capturing the inherent temporal dynamics and complex patterns in time series data.

AnomalyBERT [36] utilizes a self-supervised method, which does not require labeled data, a common scenario in real-world situations. Its innovative data degradation scheme introduces synthetic outliers, enhancing the model’s ability to detect various types of anomalies while effectively capturing temporal dependencies and contextual information using the Transformer architecture. However, the model may face challenges in training due to the complexity of the Transformer architecture and the need to balance the data degradation scheme. MAD [34] also adopts a self-supervised learning approach, eliminating the need for labeled data. However, MAD focuses on masked modeling, simplifying the training process by learning representations of normality, which reduces the complexity introduced by the integration of synthetic outliers. MAD improves upon the traditional Next Step Prediction (NSP) task by using masked inputs, allowing the model to learn from bidirectional contexts. Nevertheless, compared to NSP models, MAD is slower during the inference process, especially when masking all steps sequentially. To enhance inference speed while maintaining accuracy, CLformer [35] proposes a lightweight Transformer, enhancing local feature extraction through multi-scale causal convolution and reducing error accumulation. Additionally, the block recurrent prediction strategy decreases time and space complexity, leading to faster inference.

### 4.5. Pros and Cons

This section explores the advantages and disadvantages of prediction-based time series anomaly detection methods. CNNs excel as feature extractors for capturing local features in time series data; however, their kernel size and operational mechanisms limit their ability to capture global dependencies, which restricts their performance as a backbone for time series data. RNN-based models perform well in capturing long-term dependencies, but face scalability challenges when processing long sequences due to their sequential nature and memory limitations. GNN-based models provide deep insights into spatio-temporal dynamics, but their increased computational complexity poses additional challenges when handling large-scale or high-dimensional data, and they may not be suitable for non-graph or fuzzy graph structures. In contrast, Transformer-based Models typically outperform others in anomaly detection tasks, as they are better at handling long-range dependencies and achieving parallel processing. However, these models may require further adjustments to ensure that important contextual information is not overlooked.

## 5. Reconstruction Methods

Reconstruction-based methods use a normal dataset to train a model that attempts to encode the data into a latent space and then reconstruct the original data from this representation. Reconstruction loss is calculated by comparing the differences between the reconstructed data and the original data, as illustrated in Figure 5b. This section delves into reconstruction anomaly detection methods, focusing on four types: AE-based, VAE-based, GAN-based, Transformer-based, and Diffusion-based models.

### 5.1. AE-Based Models

As an innovative application in the field of unsupervised learning, models based on autoencoders (AE) [68] have made significant progress in the area of AD. DAGMM [37] is the pioneering model that integrates deep AE with GMM [65] for unsupervised anomaly detection. Unlike traditional techniques that perform density estimation after dimensionality reduction, DAGMM optimizes both processes simultaneously, ensuring that key information for anomaly detection is preserved. However, DAGMM relies on deep autoencoders, which face challenges in escaping local optima and require substantial computational resources. To address this issue, USAD [39] was proposed, utilizing adversarial training to enhance the stability and speed of autoencoder-based anomaly detection. The architecture of USAD enables it to train quickly and isolate anomalies effectively, making it suitable for large-scale applications. However, USAD may struggle with detecting subtle anomalies because it focuses on reconstruction error. In response to this challenge, Zhang et al. [38] introduced MSCRED, which incorporates a multi-scale convolutional recurrent encoder-decoder framework to capture temporal dependencies and inter-sensor correlations within multivariate time series data. Its approach using signature matrices and an attention mechanism allows the model to robustly diagnose anomalies of varying severity levels. However, the complexity of the model may pose challenges when dealing with extremely high-dimensional data or real-time applications. The next method NPSR, proposed by Lai et al. [40], also faces the challenge of handling the complexity of high-dimensional data. NPSR modulates the anomaly detection process by introducing a Nominality Score, balancing the detection of point and contextual anomalies. The NPSR framework includes both point-based and sequence-based reconstruction models, and its performance surpasses existing techniques, offering a theoretically grounded method for anomaly detection. Further optimization is still needed for its application in high-dimensional settings to ensure computational efficiency.

### 5.2. VAE-Based Models

Variational Autoencoders (VAEs) [69] use an encoder to map input data into a space of latent random variables, and then reconstruct the data from this space with a decoder.

LSTM-VAE [41], the former network is replaced by LSTM in VAE. However, LSTM-VAE ignores the temporal dependence between stochastic variates. Hence, OmniAnomaly [42] was proposed, the model is the first MTSAD algorithm capable of handling explicit temporal dependencies among random variables. It provides a novel method for entity-level anomaly explanation through stochastic recurrent neural networks, the objective is to learn the normal patterns of data and utilize reconstruction probability for anomaly detection. Additionally, InterFusion [43] is an unsupervised method that models both the inter-dependency and temporal dependency of MTS simultaneously. Its core idea involves modeling the normal patterns within data using a hierarchical VAE with two stochastic latent variables, each learning low-dimensional embeddings of either inter-metric or temporal aspects. Unsupervised VAE is vulnerable to the influence of anomalous inputs. SLA-VAE [44] addresses the challenge of obtaining labeled data in InterFusion through semi-supervised learning, utilizing active learning to leverage a small number of highly uncertain samples for learning and updating online system anomaly detection models. LARA [16] solves the need for online systems to quickly adapt to new distributions with a lightweight retraining approach. LARA designs the retraining process as a convex problem to prevent overfitting and ensure rapid convergence. It also has the capability to utilize historical data without the need for storage.

### 5.3. GAN-Based Models

The principle of GAN [70] is based on a framework known as adversarial training, which consists of two components: a generator designed to mimic the distribution of real data, and a discriminator tasked with distinguishing between generated and authentic data.

MAD-GAN [45] employs LSTM-RNNs as both the generator and discriminator within a GAN framework to seize the temporal relationships between time series distributions, while also taking into account the complete dataset to uncover the underlying interactions among them. MAD-GAN using standard adversarial loss suffers from gradient explosion and mode collapse. Hence, TadGAN [46] combines wasserstein loss [71] and cycle consistency loss [72] to form the final minmax objective, the former aims to match the distribution of generated time series with the data distribution of the target domain, while the latter prevents conflicting outputs between the two generators. Additionally, MIM-GAN [47] introduces an exponential information metric into the loss function of GANs to avoid local optimal solution and model collapse. It considers a discriminative reconstruction score composed of discriminative and reconstruction loss. MIM-GAN employs LSTM-based generators and discriminators to capture temporal relationships within the data. Soft-DTW [73] is used as a differentiable alternative for the reconstruction loss, and the combination of reconstruction loss and the prior probability distribution of the latent space serves as the anomaly score. DCGANs [17] can adjust the generator to produce samples closer to real data by minimizing the Soft-DTW loss. DCGANs utilize CNNs in both the generator and discriminator networks, while performing parallel computation for the reconstruction of multiple points, effectively alleviating performance bottlenecks (i.e., Mode Collapse). In complex systems, the data generated by devices exhibit complex patterns and a scarcity of labeled data, making anomaly detection a significant challenge. DAEMON [48] employs two discriminators to adversarially train an autoencoder to learn the normal patterns of time series, using reconstruction errors to identify anomalies. Its robustness is ensured through regularization of hidden variables and reconstructed data.

### 5.4. Transformer-Based Models

Due to the complex temporal characteristics (i.e., temporal dependency, inter-variable correlations, and noise) in multivariate time series, many different hybrid schemes have been proposed. TransAnomaly [50] is the first model combining Transformer and VAE for time series data. TransAnomaly enables more parallelization and reduces learning costs. TranAD [52] proposes the use of attention-based sequence encoders for rapid inference, combined with adaptive conditioning and adversarial training to enhance the stability of the model. However, the problem of over-generalization persists. MEMTO [53] introduces a novel memory module that can learn how each memory item should be updated in response to input data, adapting to diverse normal patterns. Due to the rarity of anomalies in time series data and the difficulty of establishing non-trivial associations from anomalies to the entire series, Anomaly Transformer [51] calculates association discrepancy through a new Anomaly-Attention mechanism and enhances the distinguishability between normal and abnormal points with a minimax strategy, demonstrating the potential of Transformers in capturing associations of anomaly points in time series.

The aforementioned methods are all in the time domain and do not utilize frequency domain information. Recent studies have attempted to improve the accuracy of anomaly detection by combining information from both the time and frequency domains. Dual-TF [54] proposes a Nested-Sliding Windows (NS-window) technique, where anomaly scores in the time domain are calculated within the outer window, and anomaly scores in the frequency domain are calculated within the inner window, achieving alignment of anomaly scores at the granularity of individual data points. CATCH [55] performs patching operations in the frequency domain to capture fine-grained frequency characteristics and uses a Channel Fusion Module (CFM) to perceive correlations between channels. It uses Frequency-Enhanced Point-Granularity Scoring technology [54] to calculate the anomaly score for each time point, which involves patch scanning and frequency reconstruction within the input window.

### 5.5. Diffusion-Based Models

Diffusion models perturb the observed data by gradually adding Gaussian noise [74] and then utilize a learnable transformation to recover the original data.

DiffusionAE [49] conditions the diffusion process on autoencoder reconstructions rather than the original data. DiffusionAE achieves greater robustness to varying training anomaly ratios and better handling of multiple anomaly types (i.e., point anomaly) in the dataset. The diffusion process smooths out abnormal fragment, resulting in higher reconstruction errors, which in turn improves performance. However, the training cost to adjust the information bottleneck in the reconstruction process is high. $D^{3}R$ [18] introduces a method called noise diffusion to externally manage the information bottleneck. Diffusion reframes the bottleneck by treating noise as the limiting factor and uncontaminated information as a condition. As the bottleneck is no longer inherent to the model, it can be adjusted in size without requiring retraining.

### 5.6. Pros and Cons

This section describes the advantages and limitations of the aforementioned anomaly detection models for time series data. AE-based methods automatically extract data features but cannot capture all the complexities of the data and have difficulty interpreting the meaning of reconstruction errors. VAE is adept at explicitly modeling probabilities and provide a theoretical foundation for understanding data distributions. However, the models rely on the assumption of Gaussian distributions, which may limit the expressive power of these models. GAN-based models learn complex data distributions through adversarial training, maintaining an impressive fidelity to the original data distributions. Nevertheless, they are very challenging to train due to issues like vanishing gradients [75], which can hinder model stability and convergence. Diffusion models perform well when facing noise and missing data, as they can infer missing parts during the generation process, allowing them to better understand the distribution of normal data when detecting anomalies. However, when handling the boundary between missing and observed parts, the model may exhibit inconsistencies [49]. Additionally, capturing the dynamic characteristics of time series may require further adjustments and improvements.

## 6. Contrastive Methods

Contrastive methods operate within the framework of contrastive learning, which typically involves a dual-tower model. Time series data is input into two distinct representation learning networks to generate two representation vectors. During the representation learning phase, representations of the time series are extracted from different perspectives, it is necessary to use a contrastive fusion module (such as upsampling) to align the representations to each timestep. Subsequently, a contrastive loss is employed to calculate the similarity between different representations. As illustrated in Figure 5c. The method is commonly used in unsupervised or self-supervised learning tasks. This section delves into contrastive anomaly detection methods, focusing on three types: Transformer-based, MLP-based models, LLMs-bsed.

### 6.1. LLMs-Based Models

Recently, LLMs have gained popularity in time series Analysis [76,77]. Refs. [56,58] papers share the same model name, AnomalyLLM, but they adopt different strategies to address anomaly detection issues in their respective fields. The former AnomalyLLM [56] involves pre-training a dynamic-aware encoder to generate representations of edges in dynamic graphs. This includes constructing structural-temporal subgraphs and optimizing with contrastive loss to capture the structural and temporal information of the edges. By selecting word embeddings related to dynamic graphs and clustering them into text prototypes, these text prototypes are then used to reprogram edge embeddings to align the semantics between dynamic graphs and LLMs. A prompt template is designed to encode the information of a few labeled samples into the LLM, enabling the model to adapt to various types of anomalies without updating model parameters. The latter, proposed by Liu et al. [58], introduces a knowledge distillation-based method where a student network is trained to mimic the output of a teacher network that is pre-trained on a large-scale dataset. During the testing phase, anomalies are detected when there is a significant discrepancy between the outputs of the student and teacher networks. To consolidate normal feature extraction, prototypical signals are incorporated into the student network, making it more focused on historical normal patterns. Synthetic anomalies are generated through data augmentation techniques to expand the representation gap between the two networks. Additionally, a contrastive loss is used to bring the representations of original and augmented samples in the teacher network closer together, serving as a regularization term to encourage the teacher network to capture more general patterns.

aLLM4TS [57] adapts LLMs for time series through a two-stage training strategy. Initially, it undergoes causal continuous pre-training on various time series datasets, followed by fine-tuning for multi-patch prediction in specific time series contexts. A distinctive feature of this framework is the patch-wise decoding layer, which converts individual data patches (segments) directly into time series, thereby significantly enhancing the model’s ability to master time series representations based on temporal patches.

### 6.2. MLP Mixer-Based Models

Recently, analyses of time series prediction have shown that the MLP-Mixer can effectively handle sequential data [78,79,80]. PatchAD [59] represents a departure from traditional reconstruction-based approaches. The model consists of four distinct MLP Mixers, specifically designed to achieve efficiency and light-weight using the MLP architecture. PatchAD innovatively introduces a dual-projection constraint module to alleviate potential model degradation. For practitioners and researchers seeking the latest and potentially more effective methods for MTSAD, this paper may be valuable reading material.

### 6.3. Transformer-Based Models

Existing reconstruction-methods frequently fail to handle sufficient temporal context and inadequate representation of normal patterns, hindering their effectiveness in identifying abnormal behaviors. The TRL-CPC [60] explores the integration of contrastive learning into time series analysis. In TRL-CPC, the context vectors represent observation windows in MTS. The latent representations of subsequent time steps are obtained through nonlinear transformations of these context vectors are contrasted with the latent representations of the encoder for multivariate time series to maximize positive density. RESIST [61] relies on a robust loss function based on contrastive learning to train a Transformer that models the expected behavior of network activity, without the need for an anomaly-free training subset. It automatically mitigates the impact of atypical corrupted training data to reduce their influence on training optimization. DCdetector [13] employs a contrastive learning framework to learn representations of time series data. By utilizing a dual-attention mechanism, it obtains representations of the input time series from two branches (the in-patch branch and the patch-wise branch). The contrastive loss function is defined by measuring the similarity of representations from these two different perspectives, thus acquiring permutation-invariant representations with superior discriminative capabilities.

Recent research has begun to combine reconstruction and contrastive learning to create a comprehensive integrated anomaly score that can capture deviations from expected patterns and effectively identify subtle anomalies. SimAD [62] combines a sophisticated feature extractor adept at handling extended time windows, integrates normal behavior patterns comprehensively using the EmbedPatch encoder, and introduces an innovative ContrastFusion module aimed at highlighting distribution differences between normal and abnormal data to enhance the robustness of AD. RESTAD [63] implements a Radial Basis Function (RBF) neuron layer in the Transformer model to represent a non-parametric density in the latent space. A high output from the RBF indicates similarity with normal training data. Additionally, RESTAD merges RBF similarity scores with reconstruction errors to enhance anomaly detection sensitivity.

### 6.4. Pros and Cons

This section describes the advantages and limitations of the aforementioned anomaly detection models for time series data. MLP Mixer-based methods feature a simple structure that is easy to implement and can be adapted to various data complexities by adjusting the number of layers and neurons [78]. Although MLP can capture nonlinear relationships, they may not effectively handle extremely complex data distributions. LLMs-based models have learned a wealth of semantic knowledge through pre-training on large text datasets, possessing strong representational capabilities. They are adaptable to a variety of downstream tasks and can also perform well in few-shot learning scenarios, but directly applying LLMs for TSAD is not effective. It requires the design of prompting strategies and proper fine-tuning to enhance their ability to detect and interpret anomalies [76]. Moreover, LLMs may struggle with more complex, context-related anomalies and may hallucinate when indexing and explaining anomaly points.

## 7. Datasets

This section compiles and summarizes a variety of datasets and evaluation benchmarks for MTSAD, aiming to provide comprehensive research resources for researchers in this field. These datasets include both general and specialized time series datasets that can be used for the evaluation of anomaly detection tasks, with the latter being applicable under certain assumptions or specific customizations. Each dataset or benchmark is described in detail through multiple dimensions and their inherent characteristics. In Table 2, we have collected and analyzed datasets that are well known or frequently cited in the field of MTSAD, which have been tested by a series of deep learning models.

Applications typically produce data via a sequence of data generation processes that mirror system operations or offer insights into the behavior of various entities. Anomalies frequently emerge as a consequence of irregularities in these data generation processes, highlighting unusual aspects of the systems and entities involved. Identifying such atypical traits can yield valuable insights across diverse applications. The deep learning models outlined below are categorized based on their respective application domains.

### 7.1. Astronomy

In the field of astronomy, datasets typically include various observational and simulation data, mainly used to study phenomena such as the universe, galaxies, stars, and planets [106,107]. The Ionosphere dataset [82] comes from the UCI Machine Learning Repository and is used for a binary classification task, with the goal of determining whether there are electrons present in the ionosphere based on received signals. The dataset includes individual feature information as well as ionosphere thresholds. It contains 351 samples and 33 features, where each sample represents a measurement of a radar echo signal. Various classification algorithms can be used for model training and validation when working with the Ionosphere dataset. The dataset has a high feature dimension, which may lead to the “curse of dimensionality” during model training. Additionally, handling noise and outliers in the dataset is another important challenge. The AERO [14] model can distinguish normal temporal patterns from potential anomalies in astronomical observations, effectively differentiate concurrent noise, and reduce the number of false alarms. SWAN-SF (Space Weather Analytics for Solar Flares) [83] is a multivariate time series benchmark dataset designed for the field of solar physics. The dataset contains 4075 solar active regions, spanning the 9-year operational period of the Solar Dynamics Observatory (SDO). Accurate prediction of solar flares is crucial for astronauts, space equipment, and satellite communication systems.

### 7.2. Aerospace

In the aerospace field, MTSAD datasets are typically used to monitor the performance parameters and telemetry data of aircraft, satellites, spacecraft, and other related equipment. Aerospace datasets are crucial for predicting equipment failures and ensuring mission success. By conducting in-depth analysis of this time series data, anomaly detection algorithms can be developed and tested to enhance the reliability and performance of aerospace systems [108,109]. For instance, the NASA Shuttle Valve Data [81] includes telemetry data from shuttle valves, which is used to analyze the health condition of the valves and predict potential malfunctions. The MSL (Mars Science Laboratory) dataset [25], collected by NASA, contains time-series data from 27 channels, each with 55 dimensions. The data is anonymized, standardized to a range of 0–1, and recorded every minute. Except for the telemetry values, which are continuous, most variables are binary (indicating whether commands were sent). An LSTM-based model can be used for anomaly detection in this dataset. Hundman et al. [25] proposed the LSTM-NDT, an unsupervised method for univariate time series anomaly detection, which serves as a non-parametric anomaly threshold method for NASA datasets.

### 7.3. Environmental Science

Datasets in the field of environmental science are primarily used for monitoring and managing natural resources, environmental quality, and the health of ecosystems [110,111]. For instance, the SMAP [25] provides measurements of soil moisture and temperature, which are crucial for understanding the water cycle, crop health, and climate change. By conducting in-depth analysis of this time series data, we can better understand the dynamics of environmental systems and provide data support for sustainable development. The OPPORTUNITY dataset [82] contains data from 23 body-worn sensors, 12 object-placement sensors, and 21 environmental sensors, used to measure the daily activities of four subjects. The OPPORTUNITY dataset is widely used in research in the fields of deep learning and artificial intelligence, particularly in activity recognition, and has become an important benchmark dataset. It is often utilized with CNNs and LSTMs combined with spatio-temporal feature extraction to capture complex activity patterns.

### 7.4. Internet of Things (IoT)

In the field of IoT, time series anomaly detection datasets are primarily used for monitoring and analyzing data streams generated by a vast array of interconnected devices and sensors. This allows for the timely detection of equipment failures, network anomalies, or unexpected behaviors [112]. The GECCO IoT [84] dataset, for instance, contains sensor data from smart home or industrial IoT devices, which is utilized for detecting device anomalies and system malfunctions. The CICIDS dataset [85] contains samples of various network attacks and normal traffic. It includes data from normal activities and simulated malicious attacks collected over five days. The data was generated using the CICFlowMeter tool and provides rich labeled data. Deep learning based models on the CICIDS dataset have significantly improved the accuracy of intrusion detection. Researchers have explored deep learning methods, such as multilayer perceptrons and convolutional neural networks, to enhance detection accuracy and efficiency.

In the IoT, millions of connected devices and smart sensors generate highly dynamic, large-scale, heterogeneous, and timestamped data. This timestamped data is at the core of IoT automation and has the potential to significantly impact industrial processes. Effectively detecting anomalies in time-series data and providing real-time actionable insights to drive improvements in industrial processes presents significant challenges. Liu et al. [113] proposed the AMCNN-LSTM model, which emphasizes communication efficiency and federated learning (FL) methods on devices, enabling distributed edge devices to collaboratively train an anomaly detection model. To achieve real-time anomaly detection, Nizam et al. [114] proposed a hybrid end-to-end deep anomaly detection framework that leverages CNN and LSTM networks to develop an advanced anomaly detection system. The system architecture consists of three layers: sensors and machines in the industrial setting, an edge layer for real-time data processing, and a cloud layer for offloading processing tasks. The framework is designed to accurately detect anomalies and extremely rare events in IoT streaming data.

### 7.5. Business and Finance

In the business and finance sector, time series datasets are crucial for identifying and preventing fraud, market anomalies, credit risks, and other financial issues [3]. The NAB-realAdExchange dataset [87] is part of the Numenta Anomaly Benchmark (NAB). This dataset records online advertisement click-through rates, with the metric being cost per click (CPC). It contains one file with no anomalies and another file with anomalies, designed to evaluate the performance of anomaly detection algorithms on real-time streaming data. The Credit Card dataset [88] contains records of credit card transactions, primarily used for identifying and detecting anomalous transactions, that is, potential fraudulent activities.

When dealing with financial time series involving various market risk factors, a major issue is the presence of anomalies. These anomalies can lead to calibration errors in models used for quantifying and managing risk, resulting in potential misestimations of risk. Crépey et al. [115] proposed an anomaly detection method for financial time series that combines Principal Component Analysis (PCA) and neural networks. By reducing dimensionality with PCA, this approach effectively extracts key features from high-dimensional financial time series data, simplifying the subsequent anomaly detection process. Neural networks are then applied to identify nonlinear relationships and complex patterns. To detect anomalies in more practical business scenarios in the real world, contextual information is needed for accurate prediction. Time-CAD [116] is a context-aware deep time series decomposition framework that uses deep learning techniques to decompose time series data and incorporates contextual information to improve the accuracy of anomaly detection. This method is capable of effectively detecting anomalies in business processes.

### 7.6. Cybersecurity

In the field of cybersecurity, time series anomaly detection datasets are primarily used for monitoring and analyzing network traffic, user behavior, and system logs to promptly identify security threats, intrusion attempts, and abnormal activities. The KDDCUP99 [86] is a classic cybersecurity dataset widely used for research in network anomaly detection. It contains various types of cyber attacks, such as DoS (Denial of Service), Probe, R2L (Remote to Local), U2R (User to Root), as well as traffic data from normal behavior. The Kitsune dataset [82] is a dataset used for network intrusion detection, containing both normal and anomalous network traffic data. This dataset is used to train and test deep learning models to differentiate between normal and malicious traffic. KitNET [117] is a deep learning model that uses a set of autoencoders for online network intrusion detection. This unsupervised method can learn to distinguish the characteristics of normal and anomalous network traffic without the need for expert-labeled traffic data.

### 7.7. Industrial Control Systems

In the field of Industrial Control Systems (ICS), time series datasets are commonly used for monitoring and analyzing the operational status of industrial equipment. This facilitates the timely detection of equipment failures, prediction of maintenance needs, optimization of production processes, and ensuring production safety. The WADI (Water Distribution) dataset [93], collected and open-sourced by the iTrust institute of the Singapore University of Technology and Design (SUTD), is used to simulate water treatment and distribution networks.This dataset includes sensor data and network data, covering multiple stages of the water treatment process, such as raw water intake, chemical treatment, filtration, dechlorination, and reverse osmosis. The SWaT (Secure Water Treatment) [91] is also provided by the iTrust institute of SUTD and serves as a test platform for research in the field of ISC for water treatment processes. The SWaT dataset contains sensor data from various stages of the water treatment process.

Industrial Control Systems (ICS) are widely used and are crucial to both industry and society. Failures in these systems can have severe impacts on the economy and human life. As a result, these systems have become attractive targets for both physical and cyberattacks. Kravchik et al. [118] proposed an efficient method for detecting network attacks in ICS, which combines lightweight neural networks with Principal Component Analysis (PCA). This approach aims to reduce computational resource consumption while maintaining high detection accuracy, which is particularly important in resource-constrained ICS environments. Anomaly detection is critical for preventing cybersecurity intrusions and system attacks. Many AI-based anomaly detection methods have been proposed with high detection performance, but they remain a “black box” that is difficult to interpret. Hoang et al. [119] used explainable artificial intelligence (XAI) to enhance the perspective and reliability of an LSTM-based Autoencoder-OCSVM learning model for anomaly detection in ICS. This model is capable of providing detailed information about detected anomalies, helping security teams make informed decisions.

### 7.8. Healthcare

In the healthcare domain, time series anomaly detection datasets are primarily utilized for monitoring and analyzing an individual’s physiological parameters. This allows for the timely detection of health issues, abnormalities in diseases, or malfunctions in medical equipment. For instance, the MIT-BIH Arrhythmia Database (ECG) [95] contains a vast collection of electrocardiogram (ECG) records that are used for the detection and analysis of cardiac arrhythmias. The SVDB [98] offers a range of standard and variant electrocardiogram recordings, which are employed in research on cardiac health and the detection of arrhythmias. These datasets are instrumental in advancing our understanding of heart conditions and in developing more effective diagnostic and monitoring tools for healthcare professionals.

Electrocardiogram (ECG) signals are commonly used to assess heart health. The heart, being a complex organ, can exhibit many different types of arrhythmias. Therefore, applying anomaly detection methods to analyze ECG signals can be highly beneficial. ANNet [120] utilizes LSTM networks and MLP to process ECG signals, and improves the accuracy of anomaly detection by combining feature vectors. The study also includes preprocessing steps for ECG signals, such as denoising with Discrete Wavelet Transform (DWT) and using a 60Hz notch filter to remove power line interference (PLI). Similarly, Alamr et al. [121] proposed an unsupervised transformer-based anomaly detection method. The model architecture consists of two parts: a transformer model for feature extraction and a classifier for anomaly scoring, which is used to evaluate and detect anomalies in ECG signals.

### 7.9. Server Monitoring

Time series anomaly detection datasets within the domain of server monitoring are primarily used to monitor the performance metrics of server systems to promptly identify performance bottlenecks, system failures, or security issues. For instance, SMD dataset [105] gathers a variety of server performance indicators, such as CPU usage, memory consumption, disk I/O, etc., for anomaly detection and performance analysis. Exathlon dataset [102] may collect performance data of servers under high load conditions to detect performance anomalies.

Large companies need to monitor various metrics (such as page views and revenue) of their applications and services in real time. At Microsoft, a time-series anomaly detection service has been developed to help customers continuously monitor time-series data and promptly alert potential incidents. Den et al. [122] proposed a novel algorithm based on Spectral Residual and CNN, which is the first attempt to apply the SR model, originally from the visual saliency detection domain, to time-series anomaly detection. The approach aims to provide an accurate, efficient, and general solution. Netzer et al. [123] proposed a segment-based time series anomaly detection method for monitoring machining processes. This approach detects anomalies by using subprocess-specific thresholds, enabling more precise identification and localization of abnormal behaviors in the machining process.

### 7.10. Infrastructure

Security is the foundation of sustainable urban development. The process of urban construction and operation involves a large amount of multidimensional time-series data. By detecting anomalies in MTS, decision support for urban construction and operational risk warning can be provided. Given the complexity of urban infrastructure, there is an urgent need for fast and accurate anomaly detection. Wu et al. [124] proposed a real-time anomaly detection algorithm based on an improved distance measure, called RADIM. RADIM preserves the relationships between dimensions in the multidimensional subsequences and uses an extended Frobenius norm with local weights and Euclidean distance based on multidimensional data to measure the similarity of MTS. In addition, a threshold update mechanism based on the first-order mean difference (TMFD) is designed to detect real-time anomalies by assessing deviations. This method has been applied in tunnel construction. At the same time, most current models still face challenges in perceiving high-frequency data, and the most challenging task is processing large volumes of data in an extremely short time. Liu et al. [125] proposed an anomaly detection method for high-frequency sensor data in traffic infrastructure monitoring systems based on a fine-tuned model. The researchers demonstrated that converting data into images can improve anomaly detection speed, but training deep learning models is still time-consuming. Therefore, they designed a four-stage model and compared the proposed CNN with four widely used fine-tuned CNNs using confusion matrices to rapidly detect anomalies in high-frequency data.

## 8. Conclusions and Future Direction

This article provides a comprehensive survey and review of multivariate time series anomaly detection, exploring types of anomalies and deep architectures for anomaly detection. It analyzes and organizes deep learning model architectures for anomaly detection from three perspectives, proposes a new classification of anomaly detection strategies, and discusses 46 deep learning models in depth. In addition, it compiles public datasets for multivariate time series anomaly detection, along with their respective application domains. To advance the field, the article concludes with a discussion of potential directions for future research exploration.

Discrepancy: The contrastive learning has attracted considerable attention across various domains, as it is capable of training models by contrasting positive pairs with negative pairs. Recent research has been focusing on highlighting discrepancies [59,62], and further exploration in this direction is expected.

Integration of domain knowledge: Integrating existing knowledge from the statistical domain with deep learning can further enhance the model’s capability for time series anomaly detection. FCVAE [126] selects the most useful information from the frequency domain to achieve better short-term trend construction. Future work could leverage insights from the frequency domain, combined with domain knowledge, to strengthen the analysis of time series data.

Benchmarking and evaluation metrics: The development and use of diverse public datasets that simulate real-world anomaly situations, with clear subset divisions for training and testing. At the same time, the design of intuitive, interpretable, and parameter-adjustment-free evaluation metrics to adapt to different types of time series anomaly detection.

Leveraging techniques from LLMs: Recently, LLMs have gained popularity in time series analysis [127]. LLMs exhibit exceptional generalization capabilities, demonstrating robust predictive performance even with scarce datasets. This characteristic is particularly valuable in the context of MTSAD. By leveraging multimodal knowledge, such as incorporating additional textual information to generate multimodal embeddings, LLMs effectively bridge the gap between normal and anomalous data in anomaly detection. This modeling approach enriches the anomaly detection process by providing a more comprehensive data representation, enabling the inclusion of diverse data sources and fostering more detailed and context-aware anomaly detection. Exploring LLMs in MTSAD is a promising avenue that could significantly enhance the accuracy and efficiency of detecting anomalies in multivariate time series data.

## Figures and Tables

**Table 1:** Multivariate Deep Anomaly Detection Models in Time Series. T/S: Temporal/Spatial|Values: [S: Spatial, T: Temporal, ST: Spatio-Temporal], T/F: Time/Frequency domain|Values: [T: Time, F: Frequency], LP: Learn Paradigms|Values: [Su: Supervised, Un: Unsupervised, Semi: Semi-supervised, Self: Self-supervised], Input: Model Input|Values: [P: Point, W: Window].

Model	Venue	Bonkbone	LP	S/T	T/F	Input	Code	Language
		Forecasting						
DeepAnt [22]	IEEE’2018	CNN	Un	T	T	W	✔	Pytorch
TCN-ms [23]	IOP’2019	CNN	Semi	T	T	W	–	–
TimesNet [24]	ICLR’2023	CNN	Un	T	F	W	✔	Pytorch
LSTM-NDT [25]	KDD’2018	RNN	Un	T	T	W	✔	Tensorflow
LGMAD [26]	Elsevier’2019	RNN	Semi	T	T	P	–	–
THOC [27]	NeurIPS’2020	RNN	Self	T	T	W	–	–
AD-LTI [28]	TKDE’2020	RNN	Un	T	F	P	–	–
MTAD-GAT [29]	ICDM’2020	GNN	Self	ST	T	W	✔	Tensorflow
GDN [30]	AAAI’2021	GNN	Un	S	T	W	✔	Pytorch
FuSAGNet [31]	KDD’2022	GNN	Un	ST	T	W	–	–
DVGCRN [32]	ICML’2022	GNN	Un	ST	T	W	–	–
DyGraphAD [33]	ACM’2024	GNN	Un	ST	T	W	–	–
MAD [34]	IJCNN’2022	Transformer	Self	T	T	W	–	–
CLFormer [35]	EAAI’2023	Transformer	Un	ST	T	W	–	–
AnomalyBERT [36]	ICLR’2023	Transformer	Self	T	T	W	✔	Pytorch
		Reconstruction						
DAGMM [37]	ICLR’2018	AE	Un	T	T	P	✔	Pytorch
MSCRED [38]	AAAI’2019	AE	Semi	T	T	W	✔	Tensorflow
USAD [39]	KDD’2020	AE,GAN	Un	T	T	W	✔	Pytorch
NPSR [40]	NeurIPS’2024	AE	Un	T	T	W	✔	Pytorch
LSTM-VAE [41]	IEEE’2018	VAE,RNN	Semi	T	T	P	✔	Tensorflow
OmniAnomaly [42]	KDD’2019	VAE,GRU	Semi	T	T	W	✔	Tensorflow
InterFusion [43]	KDD’2021	VAE	Un	T	T	W	✔	Tensorflow
SLA-VAE [44]	WWW’2022	VAE	Semi	T	T	W	✔	Pytorch
LARA [16]	WWW’2024	VAE	Un	T	T	W	✔	Pytorch
MAD-GAN [45]	ICANN’2019	GAN	Un	ST	T	W	✔	Tensorflow
TadGAN [46]	IEEE’2020	GAN,LSTM	Un	T	T	W	✔	Pytorch
MIM-GAN [47]	IEEE’2023	GAN,LSTM	Un	T	T	W	✔	Tensorflow
DAEMON [48]	WSDM’2023	GAN,AE	Un	T	T	W	✔	Pytorch
DCGAN [17]	AAAI’2024	GAN,CNN	Un	T	T	W	✔	Pytorch
DiffusionAE [49]	ICDMW’2023	Diffusion,AE	Un	T	T	W	✔	Pytorch
$D^{3}R$ [18]	NeurIPS’2024	Diffusion	Un	ST	F	W	✔	Pytorch
TransAnomaly [50]	CCDC’2021	Transformer,VAE	Un	T	T	W	✔	Pytorch
Anomaly Transformer [51]	ICLR’2022	Transformer	Un	T	T	W	✔	Pytorch
TranAD [52]	VLDB’2022	Transformer	Un	T	T	W	✔	Pytorch
MEMTO [53]	NeurIPS’2023	Transformer	Un	T	T	W	✔	Pytorch
Dual-TF [54]	WWW’2024	Transformer	Un	T	TF	W	✔	Pytorch
CATCH [55]	arXiv’2024	Transformer	Un	T	TF	W	✔	Pytorch
		Contrastive						
AnomalyLLM [56]	arXiv’2024	LLMs	Un	ST	T	W	✔	Pytorch
aLLM4TS [57]	ICML’2024	LLMs	Self	T	T	W	✔	Pytorch
AnomalyLLM [58]	IJCAI’2024	LLMs	Self	T	T	W	–	–
PatchAD [59]	arXiv’2024	MLP-Mixer	Un	T	T	W	✔	Pytorch
TRL-CPC [60]	Elsevier’2022	Transformer	Un	T	T	W	–	–
RESIST [61]	Springer’2022	Transformer	Un	T	T	W	–	–
Dcdetector [13]	KDD’2023	Transformer	Self	T	T	W	✔	Pytorch
SimAD [62]	arXiv’2024	Transformer	Un	T	T	W	✔	Pytorch
RESTAD [63]	arXiv’2024	Transformer	Un	T	T	W	✔	Pytorch

**Table 2:** Public dataset/benchmark used mostly for MTSAD. R/S: The source of the data, whether it is real world data or synthetic data. Samples: Individual data points or observations within a dataset. Entities: Independent observation unit or object, such as sensors, machines, devices, etc. Dims: The number of features or variables in the data. Rate: The proportion of anomalous samples within the dataset.

Datasets/Benchmark	R/S	Samples	Entities	Dims	Rate	Domain
MSL [25]	Real	132,046	27	55	10.48	Aerospace
NASA Shuttle Valve Data [81]	Real	49,097	1	9	7.0	Aerospace
IOnsphere [82]	Real	351		33	36.0	Astronomy
SWAN-SF [83]	Real	355,330	5	51	23.8	Astronomy
SMAP [25]	Real	562,800	55	25	12.83	Environmental science
OPPORTUNITY [82]	Real	36,224	24	133	3.4	Environmental science
GECCO [84]	Real	138,521	1	9	1.25	Internet of things (IoT)
CICIDS [85]	Real	170,231		72	1.28	Internet of things (IoT)
Kitsune [82]	Real	3,018,973	9	115	17.0	Cybersecurity
Http (KDDCUP99) [86]	Real	567,479		3	0.4	Cybersecurity
Smtp (KDDCUP99) [86]	Real	95,156		3	0.03	Cybersecurity
NAB-realAdExchange [87]	Real	9616	3	2		Business and finance
Creditcard [88]	Real	284,807	1	29	0.17	Business and finance
Genesis [89]	Real	16,220	1	18	0.3	Industrial control systems
GHL [90]	Synth	200,001	48	22	0.4	Industrial control systems
SWaT [91,92]	Real	946,719	1	51	11.98	Industrial control systems
WADI [93]	Real	957,372	1	123	5.99	Industrial control systems
trimSyn [38]	Synth	17,680	1	35	2.34	Industrial control systems
MSDS [94]	Real	292,860	1	10	5.37	Industrial control systems
Arrhythmia [95]	Real	452	1	274	15.0	Healthcare
MBA [96]	Real	200,000	8	2	0.14	Healthcare
Thyroid [97]	Real	3772		6	2.5	Healthcare
SVDB [98]	Real	230,400	78	2	13.6	Healthcare
Daphnet [82,99]	Real	32,594	35	3	13.2	Healthcare
Callt2 [82,100]	Real	10,080	2	2	4.1	Infrastructure
Metro [82]	Real	48,204	1	5	0.1	Infrastructure
NYC [101]	Real	17,520		3	0.57	Infrastructure
Occupancy [82]	Real	6208	2	8	28.7	Infrastructure
Exathlon [102]	Real	47,530	39	45	18.3	Server monitoring
MBD [103]	Real	8640	5	26		Server monitoring
MMS [103]	Real	4370	50	7		Server monitoring
PSM [104]	Real	132,480	1	25	27.76	Server monitoring
SMD [105]	Real	1,416,825	28	38	4.16	Server monitoring

## Data Availability

The data presented in this study are available on the source mentioned in the text.
